# Visible light-driven C−H activation and C–C coupling of methanol into ethylene glycol

**DOI:** 10.1038/s41467-018-03543-y

**Published:** 2018-03-21

**Authors:** Shunji Xie, Zebin Shen, Jiao Deng, Pu Guo, Qinghong Zhang, Haikun Zhang, Chao Ma, Zheng Jiang, Jun Cheng, Dehui Deng, Ye Wang

**Affiliations:** 1 State Key Laboratory of Physical Chemistry of Solid Surfaces, Collaborative Innovation Center of Chemistry for Energy Materials, National Engineering Laboratory for Green Chemical Productions of Alcohols, Ethers and Esters, College of Chemistry and Chemical Engineering, Xiamen University, Xiamen, 361005 China; 2 State Key Laboratory of Catalysis, Collaborative Innovation Center of Chemistry for Energy Materials, Dalian Institute of Chemical Physics, Chinese Academy of Sciences, Dalian, 116023 China; 3 Center for High Resolution Electron Microscopy, College of Materials Science and Engineering, Hunan University, Changsha, 410082 China; 4 Shanghai Synchrotron Radiation Facility, Shanghai Institute of Applied Physics, Chinese Academy of Sciences, Shanghai, 201204 China

## Abstract

The development of new methods for the direct transformation of methanol into two or multi-carbon compounds via controlled carbon–carbon coupling is a highly attractive but challenging goal. Here, we report the first visible-light-driven dehydrogenative coupling of methanol into ethylene glycol, an important chemical currently produced from petroleum. Ethylene glycol is formed with 90% selectivity and high efficiency, together with hydrogen over a molybdenum disulfide nanofoam-modified cadmium sulfide nanorod catalyst. Mechanistic studies reveal a preferential activation of C−H bond instead of O−H bond in methanol by photoexcited holes on CdS via a concerted proton–electron transfer mechanism, forming a hydroxymethyl radical (⋅CH_2_OH) that can readily desorb from catalyst surfaces for subsequent coupling. This work not only offers an alternative nonpetroleum route for the synthesis of EG but also presents a unique visible-light-driven catalytic C−H activation with the hydroxyl group in the same molecule keeping intact.

## Abstract (web summary)

Direct transformation of methanol into two- or multi-carbon compounds is extremely attractive but remains a challenge. Here, the authors report an efficient photocatalytic route to the transformation of methanol into ethylene glycol and hydrogen over a molybdenum disulfide nanofoam-modified cadmium sulfide nanorod catalyst.

## Introduction

Methanol can be derived from a variety of carbon resources, such as natural gas or shale gas, coal, biomass, and carbon dioxide, and is an abundant and renewable one-carbon (C_1_) building block^1^. Many types of chemicals can be produced from methanol, and the carbon–carbon (C–C) bond formation is the most attractive and challenging reaction in methanol chemistry. Current conversions of methanol involving C−C bond formation are restricted to dehydrative oligomerizations such as methanol-to-olefin and methanol-to-gasoline processes, which show limited selectivity to a specific product^2^, as well as methanol carbonylation^3^. The importance of methanol chemistry is increasing under the background of growing interest in the utilization of nonpetroleum carbon resources (in particular, shale gas) and carbon dioxide for sustainable production of chemicals. This trend has become a strong incentive to develop new methods or new routes for the transformation of methanol via C−C coupling with high selectivity.

Traditionally, the conversion of methanol usually involves the activation of its O−H or C−O bond. The system that can selectively activate the unreactive C–H bond of methanol with the hydroxyl group intact and form C–C bond is rare^4^. The preferential activation of inert sp^3^ α-C–H bond in an alcohol without affecting the hydroxyl group is highly challenging in synthetic chemistry and is of high academic significance^5, 6^.

Here, we present a visible-light-driven dehydrogenative coupling of methanol into ethylene glycol (EG) (Eq. 1), in which the hydroxyl group keeps intact. EG is an important chemical having a number of applications^7^. In particular, EG is widely used for the manufacture of polyesters, predominantly poly(ethylene terephthalate) (PET). The annual production of EG is >25 million metric tons and the demand for EG is expected to increase at a rate of 5% per year^8^. In the current industry, EG is primarily produced from petroleum-derived ethylene via epoxidation to ethylene oxide (EO) and the subsequent hydrolysis of EO. This multistep process suffers from low efficiency due to the low EO yield and high energy consumption^7^. The dehydrogenative coupling of methanol would offer a fascinating nonpetroleum route for sustainable production of EG.

1 $$2{\mathrm{CH}}_3{\mathrm{OH}} \to {\mathrm{HOCH}}_2{\mathrm{CH}}_2{\mathrm{OH}} + {\mathrm{H}}_2$$

We report that CdS is a unique catalyst for the conversion of methanol to EG under visible-light irradiation. The modification of CdS nanorods with MoS_2_ nanofoams further enhances the activity and EG selectivity. An EG selectivity of 90% can be obtained with a yield of 16% and a quantum yield of above 5.0%. We demonstrate that the preferential activation of C–H bond in methanol is driven by photoexcited holes via a concerted proton–electron transfer (CPET) mechanism on CdS surfaces, forming ⋅CH_2_OH radical as an intermediate for EG formation.

## Results

### Photocatalysts Efficient for Methanol Coupling to EG

It is noteworthy that Eq. 1 cannot proceed via conventional thermocatalysis because of the thermodynamic limitation (Supplementary Fig. 1). Solar-energy-driven photocatalysis is a promising strategy to realize the C−C coupling under mild conditions^9–11^. However, so far, the photocatalytic C−C coupling has been mainly limited to larger molecules such as 2,5-dihydrofuran^10^. Basically, a semiconductor with the conduction-band edge higher (more negative) than the H_2_O/H_2_ redox potential and the valence-band edge lower (more positive) than the EG/CH_3_OH redox potential may photocatalyze Eq. 1 (Supplementary Fig. 2). However, over most semiconductors investigated, instead of EG, HCHO was formed as a major carbon-based product (Table 1), suggesting that the O−H bond is easier to be activated. EG was formed on ZnS, but ZnS only worked under ultraviolet (UV) irradiation^12^ due to its large bandgap energy (3.6 eV, corresponding to *λ* = 345 nm). We discovered that CdS, a semiconductor with a bandgap energy of 2.4 eV (corresponding to *λ* = 518 nm), catalyzed the formation of EG with better selectivity under visible light. The catalytic behavior of CdS depended on its morphology (Supplementary Table 1), and CdS nanorods exhibited the best performance for EG formation among CdS samples with different morphologies (Supplementary Fig. 3). HCHO was a major by-product along with EG and H_2_. We estimated the ratio of photogenerated electrons and holes consumed in product formation by assuming Eqs. 2–4, and the value was close to 1.0 for CdS. This confirms the occurrence of reactions of Eqs. 2–4.

2 $$2{\mathrm{CH}}_3{\mathrm{OH}} + 2{\mathrm{h}}^ + \to {\mathrm{HOCH}}_2{\mathrm{CH}}_2{\mathrm{OH}} + 2{\mathrm{H}}^ +,$$

3 $${\mathrm{CH}}_3{\mathrm{OH}} + 2{\mathrm{h}}^ + \to {\mathrm{HCHO}} + 2{\mathrm{H}}^ +,$$

4 $$2{\mathrm{H}}^ + + 2{\mathrm{e}}^ - \to {\mathrm{H}}_2.$$

**Table 1:** Catalytic performances of some typical semiconductors

Catalyst	Formation rate (mmol g_cat_^−1^ h^−1^)							e^−^/h^+a^	Selectivity^b^ (%)		
	EG	HCHO	HCOOH	CO	CO_2_	H_2_	CH_4_		EG	HCHO	HCOOH
*UV-Vis light*											
TiO_2_	0	1.6	0.11	0.16	0.042	2.0	0.053	0.91	0	84	5.6
ZnO	0	3.0	0.038	0.23	0.028	3.1	0.14	0.90	0	91	1.2
g-C_3_N_4_	0	0.79	0.33	0.11	0	1.5	0.039	0.92	0	64	27
ZnS	1.3	2.2	0.067	0.083	0	3.4	0.087	0.92	54	43	1.3
*Visible light*											
ZnS	0	0	0	0	0	0	0	—	—	—	—
Cu_2_O	0	0.46	0	0	0	0.42	0	0.91	0	100	0
Bi_2_S_3_	0	0.13	0.017	0.023	0	0.19	0	0.91	0	77	10
CuS	0	0.11	0.013	0	0	0.13	0	1.0	0	89	11
CdS particle	0.28	0.40	0	0	0	0.65	0	0.95	58	42	0
CdS rod	0.46	0.38	0	0	0	0.75	0	0.90	71	29	0
MoS_2_ sheet/CdS^c,d^	6.0	2.3	0	0	0	7.5	0	0.91	84	16	0
MoS_2_ foam/CdS^c,d^	11	2.5	0	0	0	12	0	0.92	90	10	0
MoS_2_ sheet	0	0	0	0	0	0	0	—	—	—	—
MoS_2_ foam	0	0	0	0	0	0	0	—	—	—	—

Reaction conditions: solution, 76 wt% CH_3_OH + 24 wt% H_2_O, 5.0 cm^3^; atmosphere, N_2_; light source, 300-W Xe lamp; UV-Vis light, *λ* = 320–780 nm; visible light, *λ* = 420–780 nm

^a^ The ratio of electrons and holes consumed in product formation was calculated by the equation of e^–^/h^+^ = [2 × *n*(H_2_) + 2 × *n*(CH_4_)]/[2 × *n*(EG) + 2 × *n*(HCHO) + 4 × *n*(HCOOH) + 4 × *n*(CO) + 6 × *n*(CO_2_)]

^b^ Selectivity was calculated on a molar carbon basis

^c^ CdS without designation denotes the CdS nanorod

^d^ Sheet: MoS_2_ nanosheet with a content of 5.0 wt%; foam: MoS_2_ nanofoam with a content of 5.0 wt%

### Superior Performances of MoS_2_-Foam-Modified CdS Nanorods

We loaded some typical co-catalysts^13^ onto CdS nanorods to enhance the catalytic performance. Although all the co-catalysts investigated accelerated the formation of H_2_, the formation of HCHO was enhanced more significantly than that of EG, leading to lower EG selectivity in most cases (Supplementary Table 2). It is quite unique that the addition of MoS_2_ onto the CdS nanorod not only enhances the formation of H_2_ and EG but also significantly increases the EG selectivity (Table 1). Moreover, we found that MoS_2_ nanofoam is a better co-catalyst for EG formation than MoS_2_ nanosheet. The increase in the loading of MoS_2_ nanofoam from 1.0 to 5.0 wt% gradually increased the rates of H_2_ and EG formations, but did not significantly change the rate of HCHO formation (Supplementary Table 2). Thus, the EG selectivity increased upon increasing the MoS_2_ loading amount to 5.0 wt%. A too higher MoS_2_ loading (7.0 wt%) was unbeneficial to EG formation (Supplementary Table 2). The rates of EG and H_2_ formations over the 5 wt% MoS_2_ foam/CdS reached 11 and 12 mmol g_cat_^−1^ h^−1^, respectively, which were about 24 and 16 times higher than those for the CdS nanorod alone. Further, the rate of EG formation for our MoS_2_ foam/CdS catalyst under visible-light irradiation was about 1 order of magnitude higher than that for ZnS under UV-light irradiation. The EG selectivity was 90% over the MoS_2_ foam/CdS catalyst (Table 1), which was also significantly higher than that over CdS alone or ZnS.

**Fig. 1:**
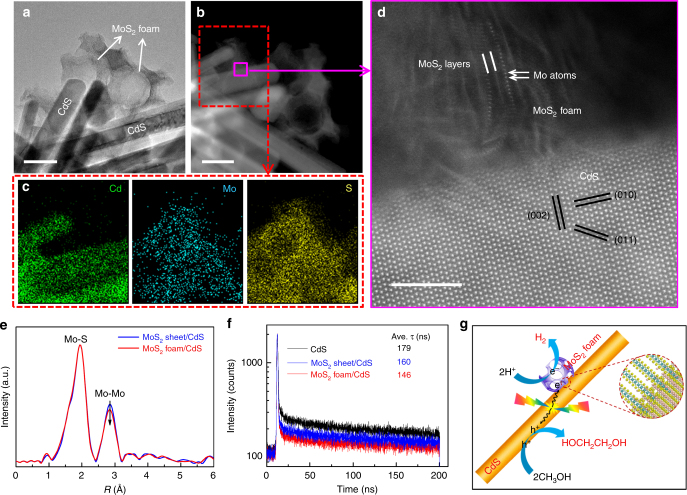
Structural and physicochemical properties of the MoS_2_ foam/CdS catalyst. **a** TEM image of MoS_2_ foam/CdS. **b** HAADF-STEM image of MoS_2_ foam/CdS. **c** Corresponding EDX maps with a red rectangle in HAADF-STEM image of **b** showing the element distribution of Cd, Mo, and S. **d** High-resolution HAADF-STEM image of MoS_2_ foam/CdS. **e** The *k*^2^-weighted EXAFS spectrum of MoS_2_ foam/CdS versus that of MoS_2_ sheet/CdS. **f** Time-resolved photoluminescence (TRPL) spectra of CdS, MoS_2_ sheet/CdS, and MoS_2_ foam/CdS. **g** Schematic illustration of MoS_2_ foam/CdS for photocatalytic synthesis of EG and H_2_ from CH_3_OH. Blue and red lines in **e** and **f** represent MoS_2_ sheet/CdS and MoS_2_ foam/CdS, respectively. The black line in **f** represents CdS. Scale bar: **a**, **b** 50 nm; **d** 5 nm

We performed characterizations to understand the origin of the significant promoting effect of MoS_2_ nanofoam. The high-resolution transmission electron microscopy (HRTEM) and high-angle annular dark-field scanning TEM (HAADF-STEM) studies revealed that the MoS_2_ foam located on the CdS rod had more edge sites and more intimate contact with CdS than the MoS_2_ sheet on CdS (Fig. 1a–1d, Supplementary Figs. 4, 5, and 6). The extended X-ray absorption fine structure (EXAFS) studies clarified that the MoS_2_ foam on CdS possessed less Mo–Mo coordination than the corresponding MoS_2_ sheet (Fig. 1e), also suggesting that the catalyst with MoS_2_ foam had more edge sites. The MoS_2_ edge sites are believed to be active sites for H_2_ evolution^14, 15^. Actually, the MoS_2_ foam/CdS catalyst also displayed a higher H_2_ formation rate than CdS and MoS_2_ sheet/CdS when a hole scavenger (Na_2_S/Na_2_SO_3_ or lactic acid) was used instead of CH_3_OH (Supplementary Table 3). On the other hand, the intimate contact between the MoS_2_ foam and CdS rod may accelerate the transfer of photogenerated charge carriers, which is also a key parameter determining the performance. The photoluminescence (PL) intensity of the emission band at ~520 nm due to the recombination of photogenerated electrons and holes decreased in the following sequence: CdS >MoS_2_ sheet/CdS >MoS_2_ foam/CdS (Supplementary Fig. 7). The PL average lifetime (ave. *τ*) derived from the time-resolved PL (TRPL) spectroscopy^16^ decreased in the same order (Fig. 1f). These results confirm the enhancement in the separation and transfer of photogenerated excitons by the presence of MoS_2_, in particular, MoS_2_ foam. The photocurrent density and the cathodic current density of linear sweep voltammetry (LSV) measurements provided further evidence for this (Supplementary Figs. 8 and 9). Therefore, MoS_2_ accelerates the photocatalytic activity for EG formation by both providing H_2_-evolution active sites and enhancing the transfer of photogenerated electrons and holes (Fig. 1g).

Furthermore, it should be reminded that the increase in the loading of MoS_2_ foam from 1.0 to 5.0 wt% increased the EG formation rate but did not significantly change the HCHO formation rate. The average pore size of MoS_2_ nanofoam derived from N_2_ physisorption is 26 nm (Supplementary Fig. 10), while the size of ⋅CH_2_OH is only 0.29 nm. Thus, the ⋅CH_2_OH radicals can easily diffuse into the mesopores of MoS_2_ nanofoam. We speculate that the mesoporous structure of MoS_2_ nanofoam may provide more probability for the coupling of the reaction intermediate to form EG. Our kinetic measurements indicate that the formation of EG is a second-order reaction, whereas the formation of HCHO is a first-order reaction (Supplementary Fig. 11). The enrichment of the intermediate inside the mesopores of MoS_2_ foam may be a reason for the enhancement in EG selectivity.

### Reaction Mechanism

**Fig. 2:**
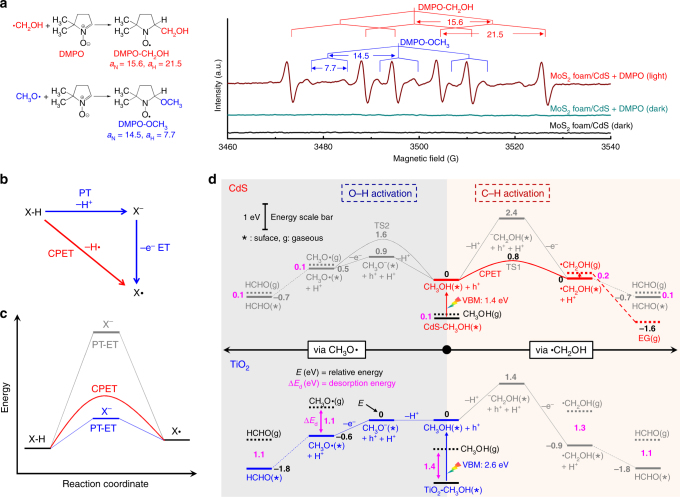
Mechanistic insights. **a** In situ ESR spectra for systems containing MoS_2_ foam/CdS catalyst in methanol aqueous solution in the presence of DMPO (a spin-trapping agent) with or without light irradiation. **b** Two possible reaction pathways. **c** Energy profile. The CPET path is favored when the stepwise PT–ET path involves a high-energy intermediate. **d** Reaction energy profiles via ⋅CH_2_OH and CH_3_O⋅ on CdS(100) and rutile TiO_2_(110)

We performed deep studies to understand the reaction mechanism for EG formation. The addition of an electron scavenger (nitrobenzene) into the system with either CdS or MoS_2_ foam/CdS catalyst stopped H_2_ formation, whereas EG formation ceased and HCHO formation rate decreased drastically after the addition of a hole scavenger (Na_2_S/Na_2_SO_3_) (Supplementary Table 4). These results provide further evidence for the reactions of Eqs. 2–4. The addition of a radical scavenger, 5,5-dimethyl-1-pyrroline-*N*-oxide (DMPO), also significantly suppressed the formation of EG and HCHO (Supplementary Table 4), suggesting that the formations of EG and HCHO proceed via radical intermediates. In situ electron spin resonance (ESR) spectroscopic studies using DMPO as a spin-trapping agent revealed the generation of the hydroxymethyl radical (⋅CH_2_OH) and methoxyl radical (CH_3_O⋅) on CdS and MoS_2_ foam/CdS catalysts (Supplementary Fig. 12 and Fig. 2a). The ⋅CH_2_OH radical should be an intermediate by C−H activation for the formation of EG, whereas the CH_3_O⋅ radical resulting from O−H activation may be responsible for HCHO formation.

What controls the preferential activation of C−H or O−H bond in methanol and the formation of intermediates over different catalysts is of paramount importance in mechanisms. To gain further insight into the reaction intermediate and the bond-activation mode, we performed density functional theory (DFT) calculations for methanol transformations on CdS and TiO_2_, two semiconductors with different product distributions; the former provided EG as a major product, while the latter predominantly catalyzed the formation of HCHO without EG (Table 1). Generally, the formation of radical intermediates through X−H (X = C or O) cleavage by accepting a hole may proceed via either a stepwise pathway, i.e., proton transfer followed by electron transfer (PT−ET) or a CPET pathway, in which proton/electron transfer takes place in a concerted manner (Fig. 2b, c). On TiO_2_ surfaces, the OH group of CH_3_OH has a moderate deprotonation energy due to the strong interaction with the ionic oxide surfaces consistent with previous studies^17^. It is therefore more likely to take a two-step PT−ET route via CH_3_O^–^ to CH_3_O⋅, eventually leading to the formation of HCHO (Fig. 2d, TiO_2_). Such a route has been demonstrated in several previous studies^18–20^. On the other hand, CH_3_OH has a negligible adsorption energy (−0.1 eV) on CdS, in contrast to that on TiO_2_ (−1.4 eV). This indicates that the OH group of CH_3_OH on CdS is difficult to deprotonate. The direct deprotonation of the C−H bond of methanol is even more difficult. It is often believed that the CPET takes place to avoid high-energy or highly unstable intermediates (Fig. 2c)^21, 22^. Our DFT studies suggest that the cleavage of the C−H bond in methanol occurs preferentially on CdS surfaces because of the following reasons. First, the formation energy of ⋅CH_2_OH is about 0.5 eV lower than that of CH_3_O⋅ (Fig. 2d, CdS). Second, assuming the nearby surface sulfur atom as a proton acceptor, the CPET driven by a hole state for the production of ⋅CH_2_OH possesses a much smaller reaction barrier (0.8 eV) than that of CH_3_O⋅ (1.6 eV) (Fig. 2d, CdS). The formed ⋅CH_2_OH intermediate has a small adsorption energy of −0.2 eV on CdS, and thus can readily desorb from the CdS surface, undertaking a thermodynamically downhill coupling to produce EG. We also found that ZnS, showing 54% EG selectivity, weakly binds ⋅CH_2_OH with an adsorption energy of −0.5 eV (Supplementary Table 5). Hence, we believe that the weak adsorption of ⋅CH_2_OH on catalyst surfaces plays a key role in the formation of EG. The strong adsorption of ⋅CH_2_OH, even if produced, on TiO_2_ (adsorption energy, −1.3 eV) and CuS (adsorption energy, −1.0 eV) will keep the intermediate on the surfaces, which then undergoes consecutive oxidation to form products such as HCHO (Fig. 2d, TiO_2_ and Supplementary Table 5).

### Process Intensification and Quantum Yield

**Fig. 3:**
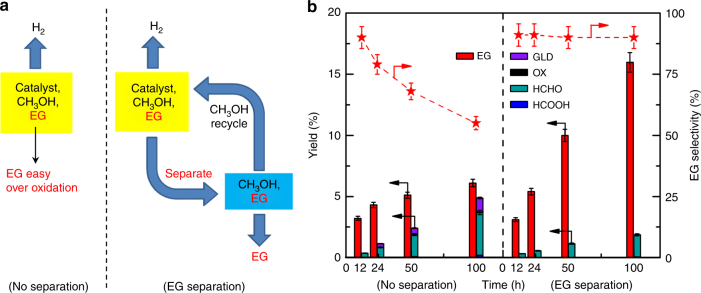
Process intensification. **a** Conventional reaction mode and process-intensified mode with EG separation. **b** Catalytic performance of MoS_2_ foam/CdS. Reaction conditions: catalyst, 20 mg; solution, 76 wt% CH_3_OH + 24 wt% H_2_O, 10 cm^3^; atmosphere, N_2_; and light source, 300-W Xe lamp, visible light (*λ* = 420–780 nm). The red star in **b** denotes EG selectivity. The experiments in each case were performed at least three times. The error bar represents the relative deviation, which is within 5%. GLD glycoaldehyde, OX oxalic acid

Considering that EG may undergo consecutive oxidation in the reaction system, we have designed a process-intensified reactor that can perform simultaneous EG separation during the reaction (Fig. 3a, Supplementary Figs. 13 and 14). In this reactor with EG separation, the MoS_2_ foam/CdS catalyst could be easily recovered and used repeatedly without significant deactivation (Supplementary Fig. 15). As compared with the conventional reaction mode, the process-intensified mode demonstrated a high EG selectivity (90%) during the longtime reaction. On the contrary, EG selectivity decreased significantly with reaction time, and many by-products such as glycoaldehyde, oxalic acid, HCHO, and HCOOH were observed in the conventional reactor (Fig. 3b, Supplementary Table 6). Therefore, EG yield could reach as high as 16% after 100 h in the reactor with EG separation.

We have measured the apparent quantum yields of EG under irradiation with different wavelengths. The quantum yield of EG was above 5.0% at wavelengths not longer than 450 nm for the MoS_2_ foam/CdS catalyst, and decreased upon increasing the wavelength (Supplementary Fig. 16). The longest wavelength suitable for EG formation (~500 nm) was found to coincide with the absorption edge of the MoS_2_ foam/CdS catalyst, which was obtained from the diffuse reflectance UV-Vis measurement. This further indicates that the EG formation is indeed driven by light.

## Discussion

The present CdS-based photocatalytic system is quite unique in the preferential activation of the C−H bond in methanol without affecting the O−H group, forming EG via ⋅CH_2_OH radical intermediate. This is achieved by photoexcited holes via the CPET mechanism on CdS surfaces. The weak adsorption of CH_3_OH and ⋅CH_2_OH intermediate on CdS decreases the possibility of O−H bond activation, which is a case on TiO_2_, and enables the facile desorption of ⋅CH_2_OH from catalyst surfaces for subsequent C−C coupling. The loading of MoS_2_ nanofoam with abundant edge sites significantly improves the formation of H_2_ and the overall activity. The EG selectivity is also enhanced probably because of the enriching effect of mesoporous nanofoam. The high selectivity of EG (90%) can be sustained in the long-term reaction by using a process-intensified reactor with EG-separation capability. The present visible-light-driven methanol transformation not only offers an atom-efficient method for the synthesis of EG under mild conditions, but also opens up a new avenue for preferential C–H bond activation without affecting other functional groups in the same molecule.

## Methods

### Synthesis of CdS Nanorods

CdS nanorods were synthesized by a modified solvothermal method^23^. Typically, 4.62 g of CdCl_2_·2.5H_2_O and 4.62 g of CH_4_N_2_S were dissolved in 60 mL of ethylenediamine. Then, the mixture was transferred to a Teflon-lined autoclave and was maintained at 160 °C for >24 h. After cooling down to room temperature, the resulting yellow solid products were collected by centrifugation, and washed with distilled water and ethanol three times. The product was then dried at 60 °C.

### Synthesis of MoS_2_ Nanofoam and Nanosheet

MoS_2_ nanofoam and nanosheet were synthesized by procedures reported previously^15^. For the synthesis of nanofoam, 0.4 g of (NH_4_)_6_Mo_7_O_24_·4H_2_O and 1.6 g of SiO_2_ nanospheres (30 wt% SiO_2_ in EG, Alfa Aesar) were first dispersed in 20 mL of deionized water. After removing the solvent, the obtained powder reacted with 0.8 g of CH_4_N_2_S at 400 °C for 4 h. The obtained product was treated in hydrofluoric acid aqueous solution under room temperature, followed by washing with deionized water and drying. For the synthesis of the nanosheet, 0.9 g of (NH_4_)_6_Mo_7_O_24_·4H_2_O was dissolved in 20 mL of deionized water and was then reacted with 10 mL of carbon disulfide at 400 °C for 4 h. The final product was obtained by treating in saturated NaOH aqueous solution at 60 °C, followed by washing with deionized water and drying.

### Preparation of MoS_2_ Foam/CdS and MoS_2_ Sheet/CdS Catalysts

A series of MoS_2_/CdS nanocomposites were prepared by an ultrasonic method^24^. For example, for the preparation of the MoS_2_ foam/CdS catalyst, MoS_2_ nanofoam (5.0 mg) was first ultrasonically dispersed in *N*,*N*-dimethylformamide (DMF,10 mL) in a flask for 3 h at room temperature. Then, CdS nanorods (100 mg) were added to the suspension. The mixture was further subjected to ultrasonic treatment for another 2 h to achieve close contact between MoS_2_ and CdS. The MoS_2_/CdS nanocomposite was collected by centrifugation and washed with deionized water and ethanol, followed by drying at 60 °C.

### Catalytic Reaction

Photocatalytic reactions were carried out in a sealed quartz-tube reactor (volume, 20 mL). The visible (Vis) light source was a 300-W Xe lamp with a UV cutoff filter (420–780 nm). The UV-Vis light (320–780 nm) irradiation without using the UV cutoff filter was also applied to some catalysts. The solid catalyst powder (10 mg) was ultrasonically dispersed in 5.0 mL of mixed solution containing 76 wt% CH_3_OH and 24 wt% H_2_O. Then, the reactor was evacuated and filled with high-purity (99.999%) nitrogen. The photocatalytic reaction was carried out at room temperature typically for 12 h. After the reaction, the liquid products were analyzed by high-performance liquid chromatography (HPLC, Shimadzu LC-20A) with refractive index and UV detectors together with a Shodex SUGARSH-1011 column (8 × 300 mm) using a dilute H_2_SO_4_ aqueous solution as the mobile phase. H_2_, CH_4_, CO, and CO_2_ were analyzed by an Agilent Micro GC3000 equipped with a molecular sieve 5A column and a high-sensitivity thermal conductivity detector. The relative deviation of detection was 4% for gas chromatography and 3% for liquid chromatography.

We measured the apparent quantum yields by using light at different wavelengths for the photocatalytic conversion of CH_3_OH to EG over the 5% MoS_2_ foam/CdS catalyst. The apparent quantum yield (*η*) for the formation of EG was calculated using the following equation:

5 $$\eta = \left[ {2{{n}}\left( {\mathrm{EG}} \right) \times {{N}}_{\mathrm{A}}} \right]/\left[ {I\left( {{\mathrm{Wcm}}^{ - 2}} \right) \times {{S}}\left( {{\mathrm{cm}}^{\mathrm{2}}} \right) \times {{t}}\left( {\mathrm{s}} \right)/{{E}}_\lambda \left( {\mathrm{J}} \right)} \right] \times 100\%,$$

where *n*(EG), *N*_A_, *I*, *S*, and *t* represent the molar amount of EG, Avogadro’s constant, light intensity, irradiation area, and reaction time, respectively. *E*_*λ*_ can be calculated using *hc*/*λ* (*λ* = 380, 420, 450, 475, 500, 550, or 600 nm).

Photocatalytic reactions with EG-separation mode (Supplementary Fig. 13) were carried out in the reactor shown in Supplementary Fig. 14. The light source was a 300-W Xe lamp with a UV cutoff filter (420–780 nm). The solid catalyst (20 mg) was ultrasonically dispersed in 10 mL of mixed solution containing 76 wt% CH_3_OH and 24 wt% H_2_O. Then, the reactor was evacuated and filled with nitrogen. The photocatalytic reaction was performed at room temperature. After the reaction, the liquid and gaseous products were also analyzed by HPLC and Micro GC.

### Characterization

The photocatalysts or photocatalytic systems were characterized by scanning electron microscopy, TEM, high-resolution HAADF-STEM, energy-dispersive X-ray spectroscopy mapping, three-dimensional tomography, steady-state and TRPL spectroscopy, EXAFS spectroscopy, ESR spectroscopy, LSV, N_2_ physisorption, diffuse reflectance UV-Vis spectroscopy, and photoelectrochemical measurements. The details of these techniques were described in Supplementary Information.

The other experimental and computational methods are displayed in Supplementary Information.

### Data Availability

The data that support the findings of this study are available from the corresponding authors upon a reasonable request.

## Electronic supplementary material

Supplementary Information(PDF 3154 kb)

Peer Review File(PDF 183 kb)

## References

[1] Olah GA (2013). Towards oil independence through renewable methanol chemistry. Angew. Chem. Int. Ed. Engl..

[2] Olsbye U (2012). Conversion of methanol to hydrocarbons: how zeolite cavity and pore size controls product selectivity. Angew. Chem. Int. Ed. Engl..

[3] Maitlis PM, Haynes A, Sunley GJ, Howard MJ (1996). Methanol carbonylation revisited: thirty years on. J. Chem. Soc. Dalton Trans..

[4] Moran J, Preetz A, Mesch RA, Krische MJ (2011). Iridium-catalysed direct C–C coupling of methanol and allenes. Nat. Chem..

[5] Zhang S, Zhang F, Tu Y (2011). Direct Sp^3^ α-C–H activation and functionalization of alcohol and ether. Chem. Soc. Rev..

[6] Cheng JK, Loh TP (2015). Copper-and cobalt-catalyzed direct coupling of sp^3^ α-carbon of alcohols with alkenes and hydroperoxides. J. Am. Chem. Soc..

[7] Yue H, Zhao Y, Ma X, Gong J (2012). Ethylene glycol: properties, synthesis, and applications. Chem. Soc. Rev..

[8] Zheng M, Pang J, Sun R, Wang A, Zhang T (2017). Selectivity control for cellulose to diols: dancing on eggs. ACS Catal..

[9] Schultz DM, Yoon TP (2014). Solar synthesis: prospects in visible light photocatalysis. Science.

[10] Kisch H (2013). Semiconductor photocatalysis—mechanistic and synthetic aspects. Angew. Chem. Int. Ed. Engl..

[11] Fagnoni M, Dondi D, Ravelli D, Albini A (2007). Photocatalysis for the formation of the C−C bond. Chem. Rev..

[12] Yanagida S, Azuma T, Kawakami H, Kizumoto H, Sakurai H (1984). Photocatalytic carbon–carbon bond formation with concurrent hydrogen evolution on colloidal zinc sulphide. J. Chem. Soc. Chem. Commun..

[13] Yang J, Wang D, Han H, Li C (2013). Roles of cocatalysts in photocatalysis and photoelectrocatalysis. Acc. Chem. Res..

[14] Jaramillo TF (2007). Identification of active edge sites for electrochemical H_2_ evolution from MoS_2_ nanocatalysts. Science.

[15] Deng J (2017). Multiscale structural and electronic control of molybdenum disulfide foam for highly efficient hydrogen production. Nat. Commun..

[16] Sun Z, Zheng H, Li J, Du P (2015). Extraordinarily efficient photocatalytic hydrogen evolution in water using semiconductor nanorods integrated with crystalline Ni_2_P cocatalysts. Energy Environ. Sci..

[17] Cheng J, Sprik M (2010). Acidity of the aqueous rutile TiO_2_ (110) surface from density functional theory based molecular dynamics. J. Chem. Theory Comput..

[18] Zhang J, Peng C, Wang H, Hu P (2017). Identifying the role of photogenerated holes in photocatalytic methanol dissociation on rutile TiO_2_ (110). ACS Catal..

[19] Guo Q (2012). Stepwise photocatalytic dissociation of methanol and water on TiO_2_ (110). J. Am. Chem. Soc..

[20] Cheng J, Liu X, Kattirtzi JA, VandeVondele J, Sprik M (2014). Aligning electronic and protonic energy levels of proton-coupled electron transfer in water oxidation on aqueous TiO_2_. Angew. Chem. Int. Ed. Engl..

[21] Warren JJ, Tronic TA, Mayer JM (2010). Thermochemistry of proton-coupled electron transfer reagents and its implications. Chem. Rev..

[22] Schrauben JN (2012). Titanium and zinc oxide nanoparticles are proton-coupled electron transfer agents. Science.

[23] Jiang D, Sun Z, Jia H, Lu D, Du P (2016). A cocatalyst-free CdS nanorod/ZnS nanoparticle composite for high-performance visible-light-driven hydrogen production from water. J. Mater. Chem. A.

[24] He J (2016). CdS nanowires decorated with ultrathin MoS_2_ nanosheets as an efficient photocatalyst for hydrogen evolution. ChemSusChem.

