# Supplementary material for: Visible light-driven C−H activation and C–C coupling of methanol into ethylene glycol
Source: Nat Commun. 2018 Mar 21;9:1181. doi: 10.1038/s41467-018-03543-y (PMC5862904; doi:10.1038/s41467-018-03543-y)
Supplement: Supplementary file 1 — Supplementary Information(PDF 3154 kb) [file 41467_2018_3543_MOESM1_ESM.pdf]

Supplementary Information

Visible light-driven C−H activation and C–C coupling of methanol into ethylene glycol

Xie et al.

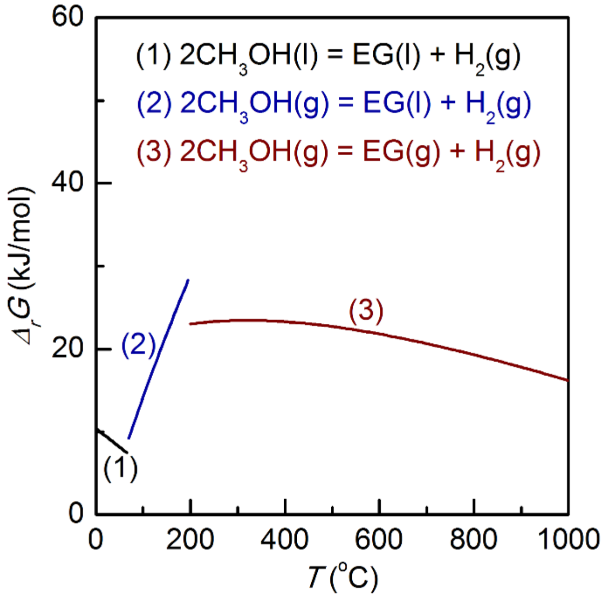

Supplementary Figure 1. Gibbs free energy changes for the dehydrogenative coupling of methanol to EG.

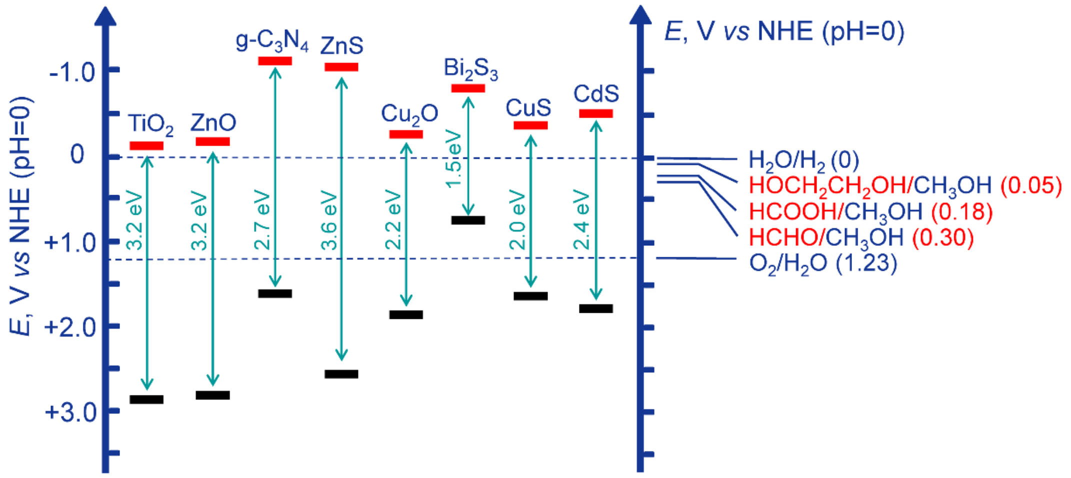

Supplementary Figure 2. Energy levels of several related redox couples and band-edge positions of some semiconductors. NHE represents the normal hydrogen electrode.

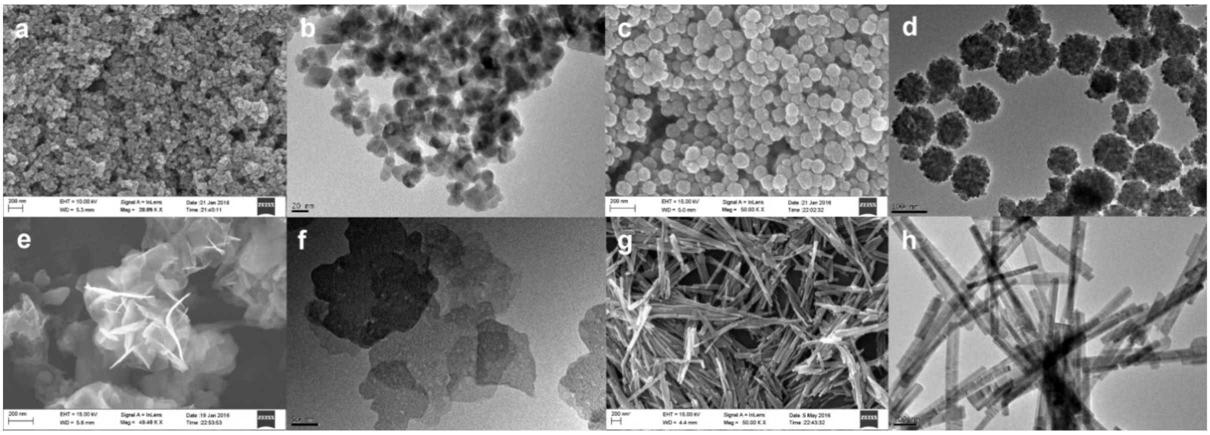

Supplementary Figure 3. Scanning electron microscopy (SEM) and transmission electron microscopy (TEM) images for CdS samples with different morphologies. **a, b,** CdS nanoparticles; **c, d,** CdS nanospheres; **e, f,** CdS nanosheets; **g, h,** CdS nanorods. Scale bar: **a**, **c, e, g** 200 nm; **b** 20 nm; **d, h** 100 nm; **f** 50 nm.

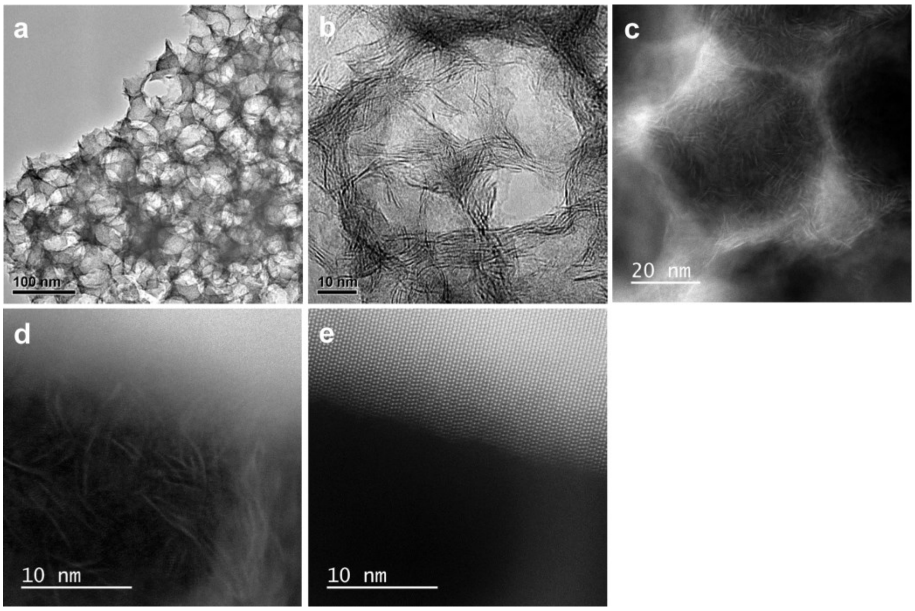

Supplementary Figure 4. High-resolution transmission electron microscopy (HRTEM) and High-resolution high-angle annular dark-field scanning transmission electron microscopy (HRHAADF-STEM) images of MoS_2 nanofoam and MoS_2-foam/CdS. **a, b,** HRTEM images of MoS_2 nanofoam; **c,** HRHAADF-STEM image of MoS_2 nanofoam; **d, e,** HRHAADF-STEM images of MoS_2-foam/CdS. Scale bar: **a** 100 nm; **b, d, e** 10 nm; **c** 20 nm.

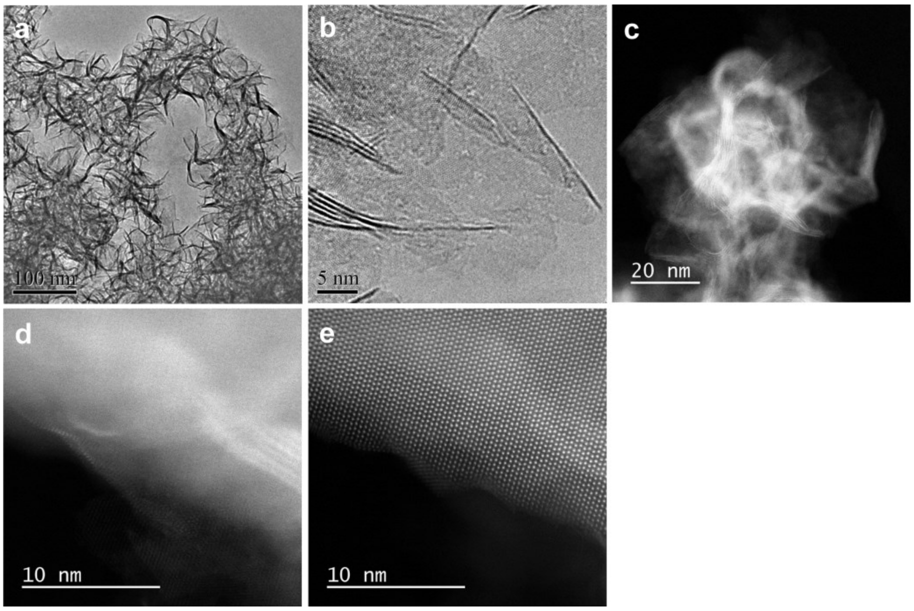

Supplementary Figure 5. HRTEM and HRHAADF-STEM images of MoS_2 nanosheet and MoS_2-sheet/CdS. **a, b,** HRTEM images of MoS_2 nanosheet; **c,** HRHAADF-STEM image of MoS_2 nanosheet; **d, e,** HRHAADF-STEM images of MoS_2-sheet/CdS. Scale bar: **a** 100 nm; **b** 5 nm; **c** 20 nm; **d, e** 10 nm.

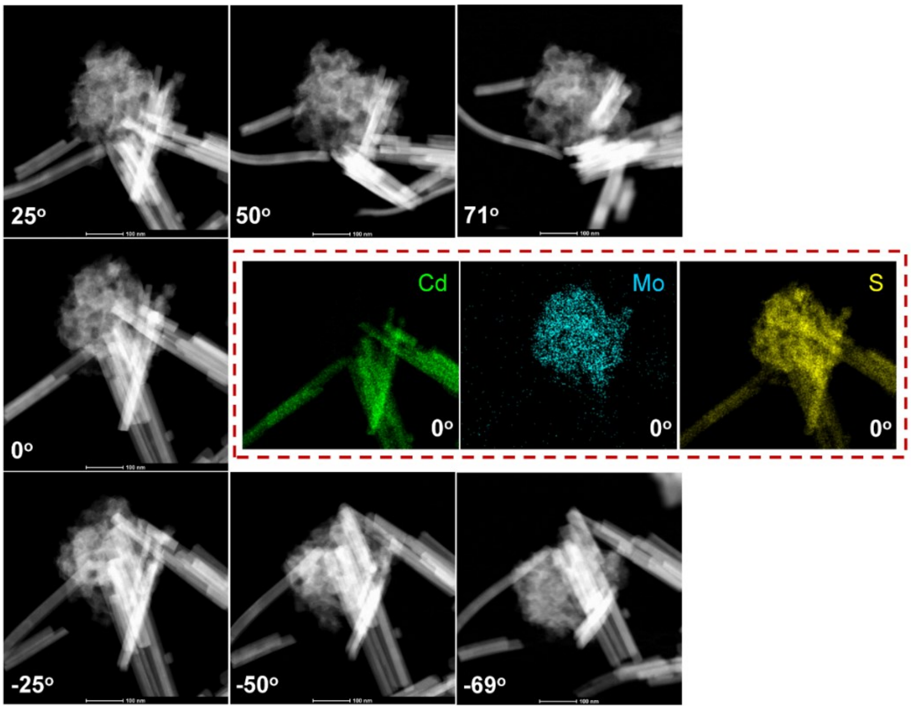

Supplementary Figure 6. HAADF-STEM images with different tilting angles and energy-dispersive X-ray spectroscopy (EDX) maps (red rectangle) of MoS_2-foam/CdS at tilting angle of 0°. Scale bar: 100 nm.

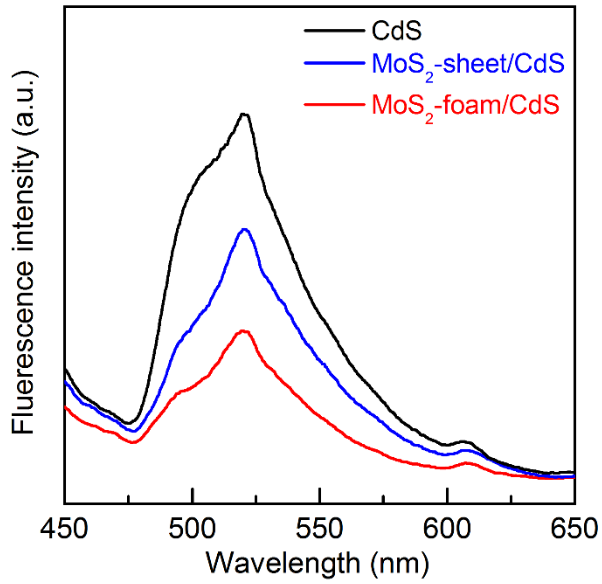

Supplementary Figure 7. Steady-state photoluminescence emission spectra under 405 nm excitation for CdS, MoS_2-sheet/CdS and MoS_2-foam/CdS.

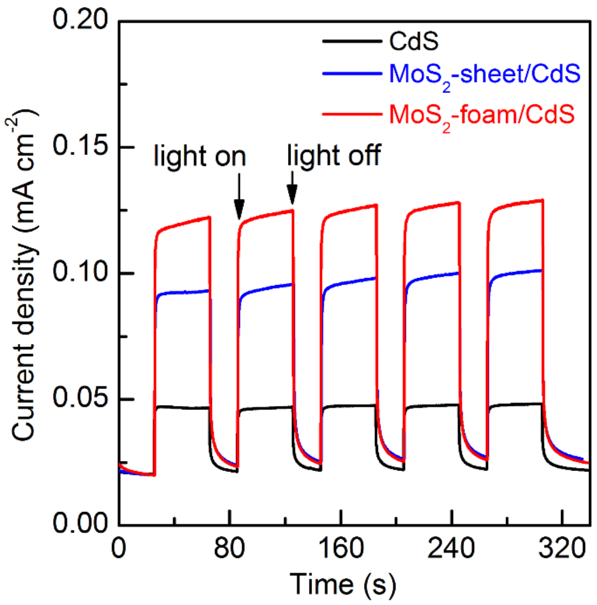

Supplementary Figure 8. Transient photocurrent responses for CdS, MoS_2-sheet/CdS and MoS_2-foam/CdS.

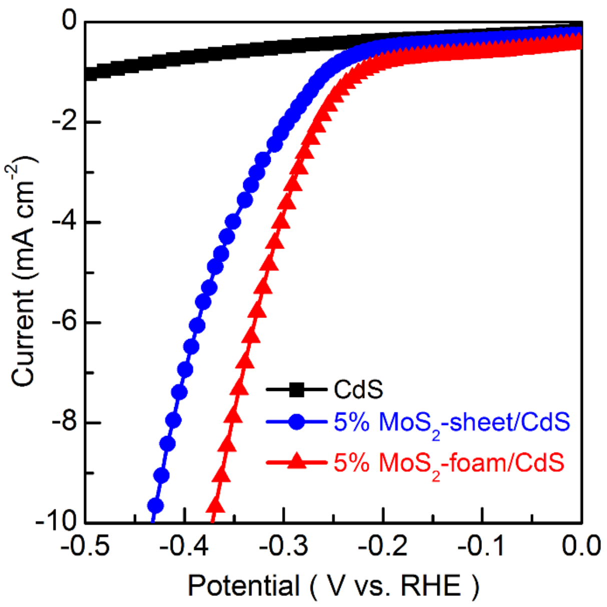

Supplementary Figure 9. Linear sweep voltammetry (LSV) curves for CdS, MoS_2-sheet/CdS and MoS_2-foam/CdS.

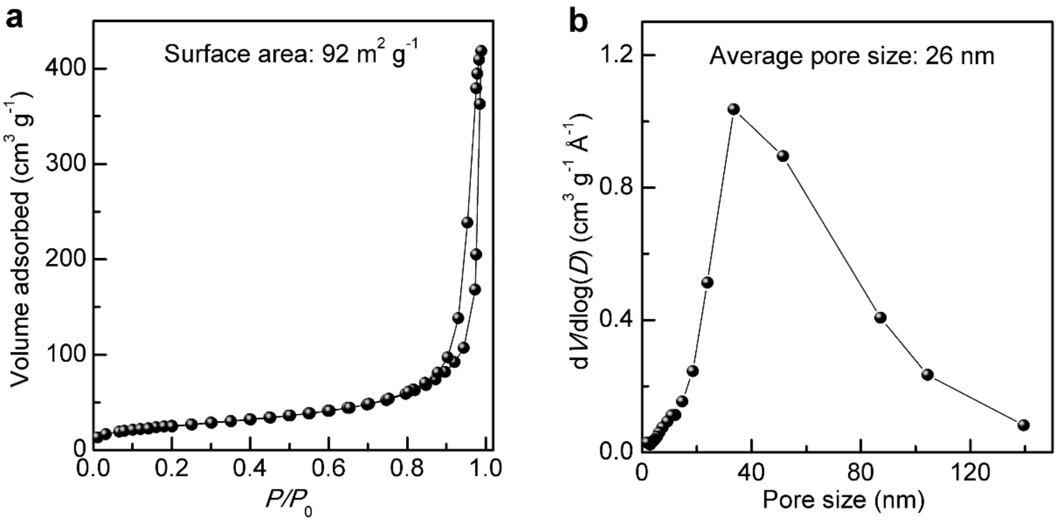

Supplementary Figure 10. a, N_2 adsorption-desorption isotherm for MoS_2-foam after sonication. **b,** The pore-diameter distribution.

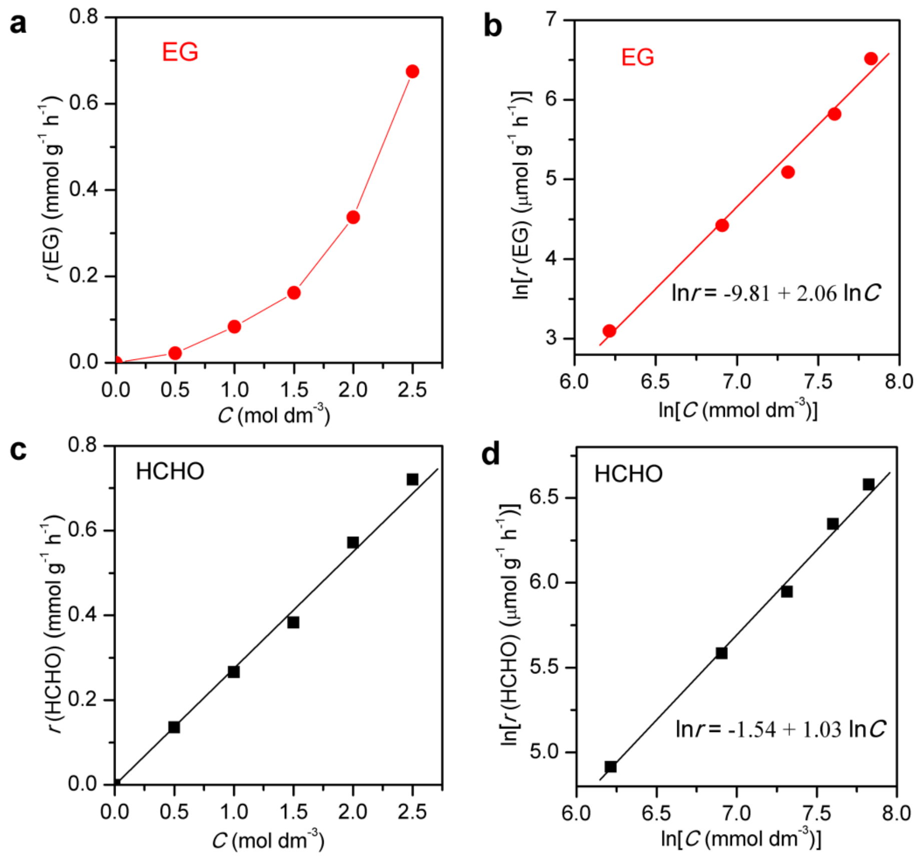

Supplementary Figure 11. Product formation rates versus CH_3OH concentration in photocatalytic conversion of CH_3OH over the MoS_2-foam/CdS catalyst. **a, b,** EG; **c, d,** HCHO. **a, c,** rate versus HCHO concentration. **b, d,** logarithm of rates versus logarithm of CH_3OH concentration. Reaction conditions: catalyst, 10 mg; solution, $CH_3OH + H_2O$, 5.0 cm^3; atmosphere, N_2; light source, 300 W Xe lamp, visible light (λ = 420-780 nm); irradiation time, 12 h. The concentration of CH_3OH was controlled at $\leq$ 2.5 mol dm^{-3}.

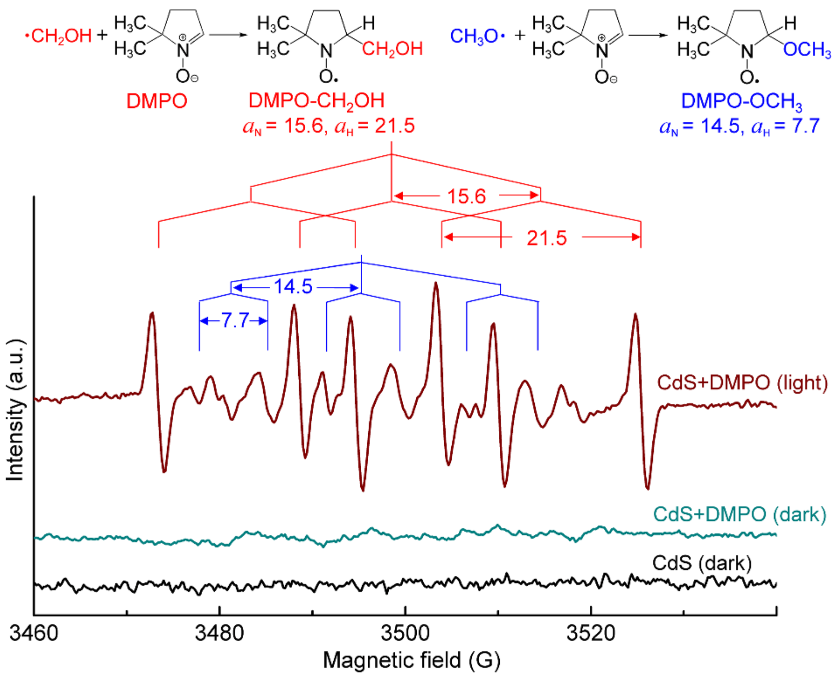

Supplementary Figure 12. In situ electron spin resonance (ESR) spectra for systems containing CdS in methanol aqueous solution in the presence of DMPO (a spin-trapping agent) with or without light irradiation.

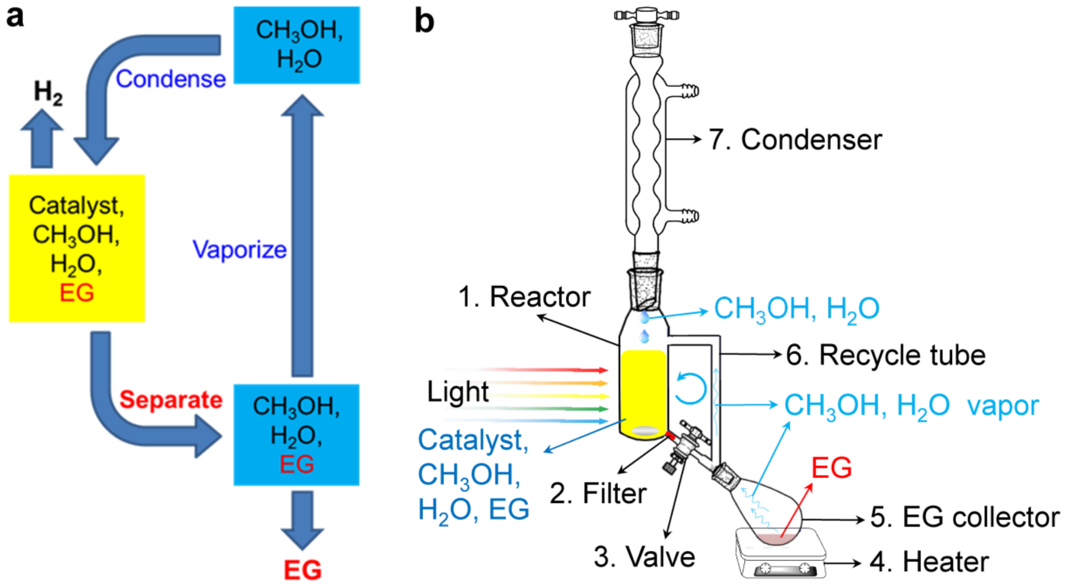

Supplementary Figure 13. a, Schematic illustration of EG-separation reaction mode. **b,** Reactor with EG separation. The photocatalytic reaction was carried out in the reactor (1). The reaction solution and the catalyst were separated by the filter (2). The valve (3) was used to control the flow rate. The reaction solution was heated by heater (4). EG remained in the EG collector (5), and at the same time, CH_3OH/H_2O was vaporized and retuned back to the reactor (1) through the recycle tube (6). The conventional reaction without EG separation was carried out with the valve (3) closed.

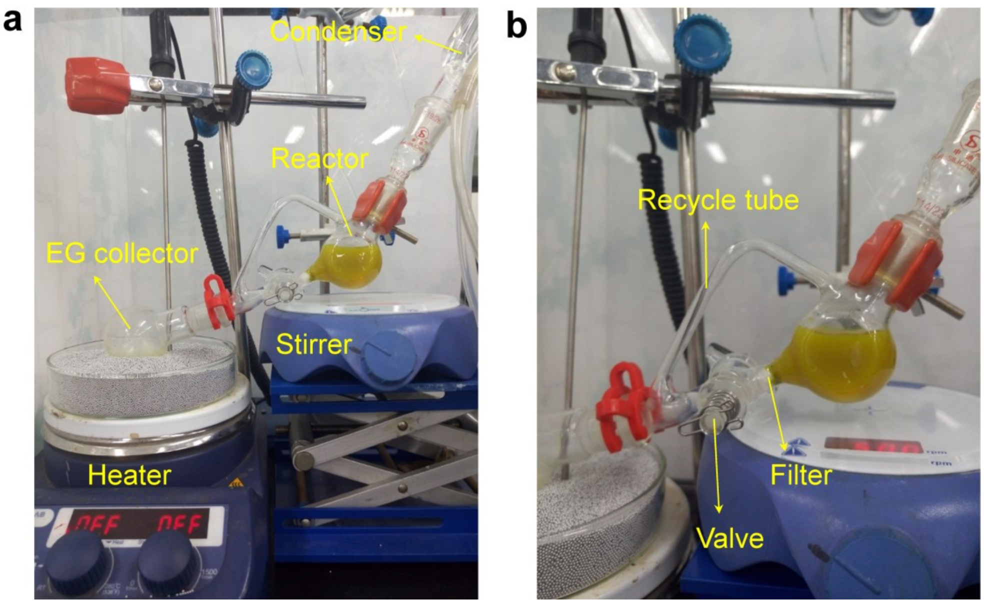

Supplementary Figure 14. Pictures of the photocatalytic reactor with EG separation. **a,** Image of the equipment. **b,** Image of reactor part.

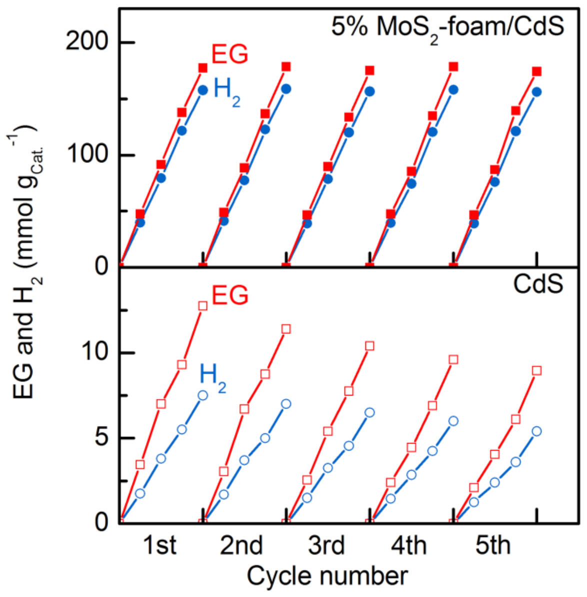

Supplementary Figure 15. Repeated uses of CdS and MoS_2-foam/CdS with EG separation reaction mode. Reaction conditions: catalyst, 20 mg; solution, 76 wt% CH_3OH + 24 wt% H_2O, 10 cm^3; atmosphere, N_2; light source, 300 W Xe lamp, visible light (λ = 420-780 nm); irradiation time, 12 h for each cycle.

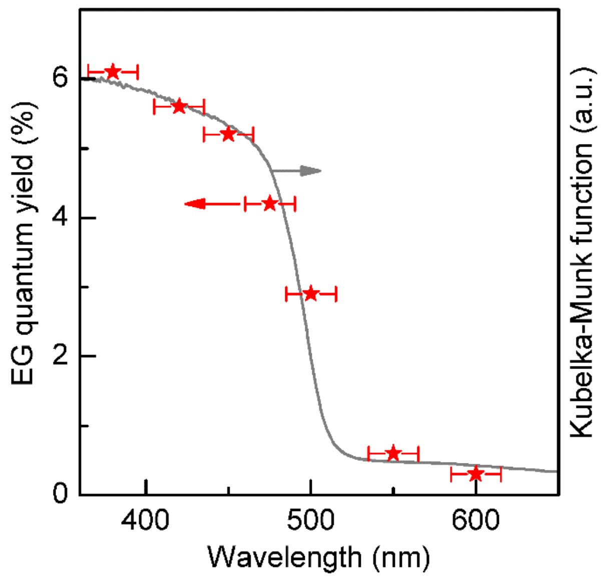

Supplementary Figure 16. Quantum yields of EG at different wavelengths and diffuse reflectance UV-vis spectrum for the 5% MoS_2-foam/CdS catalyst. Reaction conditions: catalyst, 10 mg; solution, 76 wt% CH_3OH + 24 wt% H_2O, 5.0 cm^3; atmosphere, N_2; light source, 300 W Xe lamp with different band-pass filters, error bars showing the deviation of the wavelengths ($\Delta\lambda$ = ± 15 nm); irradiation time, 6 h.

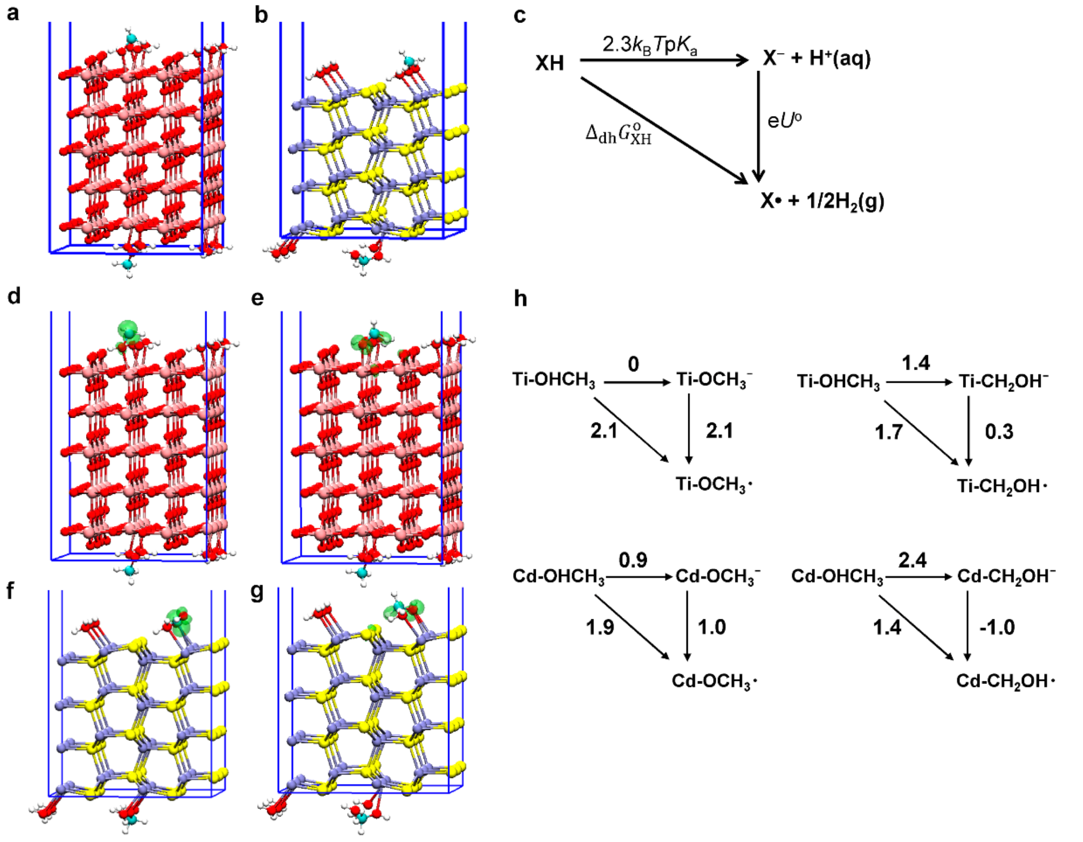

Supplementary Figure 17. Density functional theory (DFT) calculations. **a, b,** Adsorption structures of methanol molecule on the rutile TiO_2(110) (**a**) and CdS(100) (**b**) with the adsorption one monolayer water molecules. The methanol and water molecules are placed at both sides of the slabs to minimize the internal dipoles within the slabs. Atoms in pink, red, iceblue, yellow, white and cyan represent Ti, O, Cd, S, H and C, respectively. **c,** Acid dissociation (horizontal), oxidation (vertical) and dehydrogenation (diagonal) reactions in proton and electron transfer triangle. The energies are denoted as $2.3k_BTpK_a$ for deprotonation, eU^o for oxidation and $\Delta_{dh}G^o$ for dehydrogenation *vs* standard hydrogen electrode (SHE), respectively. **d, e, f, g,** Calculated structures of the most stable configurations of radical intermediates in methanol oxidation on rutile TiO_2(110) and CdS(100). (**d**) •CH_2OH with oxygen atom bonded with surface Ti; (**e**) CH_3O• with oxygen atom bonded with surface Ti; (**f**) •CH_2OH with carbon atom bonded with surface Cd; (**g**) CH_3O• with oxygen atom bonded with surface Cd. The spin densities of the surface radicals are visualized by green iso-surfaces with a density of 0.01. **h,** The energetics of the proton and electron transfer steps in methanol oxidation on rutile TiO_2 (110) and CdS (100). The energies are in eV.

Supplementary Table 1 Catalytic performances of CdS samples with different morphologies

Catalyst	Formation rate (mmol g_{cat}^{-1} h^{-1})						e^-/h^+	Selectivity* (%)		
	EG	HCHO	HCOOH	CO	CO_2	H_2		EG	HCHO	HCOOH
CdS nanoparticle	0.28	0.40	0	0	0	0.65	0.95	58	42	0
CdS nanosphere	0.37	0.38	0	0	0	0.70	0.93	66	34	0
CdS nanosheet	0.33	0.40	0.013	0	0	0.68	0.92	62	37	1.2
CdS nanorod	0.46	0.38	0	0	0	0.75	0.90	71	29	0

Reaction conditions: catalyst, 10 mg; solution, 76 wt% CH_3OH + 24 wt% H_2O, 5.0 cm^3; atmosphere, N_2; light source, 300 W Xe lamp, visible light (λ = 420-780 nm); irradiation time, 12 h. *The selectivity was calculated on a molar carbon basis.

Supplementary Table 2 Catalytic performances of CdS nanorods with different co-catalysts

Catalyst	Formation rate (mmol $g_{Cat.}^{-1}$ h^{-1})						e^-/h^+	Selectivity* (%)		
	EG	HCHO	HCOOH	CO	CO_2	H_2		EG	HCHO	HCOOH
CdS	0.46	0.38	0	0	0	0.75	0.90	71	29	0
0.5% Pt/CdS	0.38	1.2	0.28	0	0	1.9	0.91	34	53	13
0.5% Pd/CdS	0.79	0.92	0	0	0	1.8	1.1	63	37	0
5.0% NiO_x/CdS	0.63	0.79	0	0	0	1.3	0.91	61	39	0
1.0% MoS_2-foam/CdS	4.3	2.5	0	0	0	6.1	0.90	78	22	0
3.0% MoS_2-foam/CdS	8.0	2.1	0	0	0	9.1	0.91	88	12	0
5.0% MoS_2-foam/CdS	11	2.5	0	0	0	12	0.92	90	10	0
7.0% MoS_2-foam/CdS	8.6	2.1	0	0	0	9.7	0.90	89	11	0

Reaction conditions: catalyst, 10 mg; solution, 76 wt% CH_3OH + 24 wt% H_2O, 5.0 cm^3; atmosphere, N_2; light source, 300 W Xe lamp, visible light (λ = 420-780 nm); irradiation time, 12 h. *The selectivity was calculated on a molar carbon basis.

Supplementary Table 3 Control experiments of using hole scavenger instead of reactant for CdS and MoS_2/CdS catalyst

Catalyst	Reactant or hole scavenger	H_2 evolution rate (mmol g_{cat}^{-1} h^{-1})
CdS	76 wt% CH_3OH	0.8
5.0% MoS_2-sheet/CdS	76 wt% CH_3OH	7.5
5.0% MoS_2-foam/CdS	76 wt% CH_3OH	12
CdS	0.5 M Na_2S/Na_2SO_3	3.5
5.0% MoS_2-sheet/CdS	0.5 M Na_2S/Na_2SO_3	51
5.0% MoS_2-foam/CdS	0.5 M Na_2S/Na_2SO_3	73
CdS	0.5 M lactic acid	4.1
5.0% MoS_2-sheet/CdS	0.5 M lactic acid	59
5.0% MoS_2-foam/CdS	0.5 M lactic acid	82

Reaction conditions: catalyst, 10 mg; aqueous solution, 5.0 cm^3; atmosphere, N_2; light source, 300 W Xe lamp, visible light (λ = 420-780 nm); irradiation time, 12 h.

Supplementary Table 4 Control experiments for methanol conversion in the presence of different scavengers

Catalyst	Scavenger	Formation rate (mmol g_{cat}^{-1} h^{-1})				Selectivity† (%)		
		EG	HCHO	HCOOH	H_2	EG	HCHO	HCOOH
CdS	none	0.46	0.38	0	0.75	71	29	0
CdS*	none	0	0	0	0	0	0	0
CdS	0.1 M $C_6H_5NO_2$	0.75	0.63	0.017	0	70	29	0.78
CdS	0.1 M Na_2S/Na_2SO_3	0	0.054	0.021	3.1	0	72	28
CdS	0.1 M DMPO	0.075	0.071	0	0.68	68	32	0
MoS_2-foam/CdS	none	11	2.5	0	12	90	10	0
MoS_2-foam/CdS	none	0	0	0	0	0	0	0
MoS_2-foam/CdS	0.1 M $C_6H_5NO_2$	12	2.8	0.79	0	87	10	2.8
MoS_2-foam/CdS	0.1 M Na_2S/Na_2SO_3	0	0.22	0.046	45	0	83	17
MoS_2-foam/CdS	0.1 M DMPO	0.96	0.75	0	4.9	72	28	0

Reaction conditions: catalyst, 10 mg; solution, 76 wt% CH_3OH + 24 wt% H_2O, 5.0 cm^3; atmosphere, N_2; light source, 300 W Xe lamp, visible light (λ = 420-780 nm); irradiation time, 12 h. *Dark. †The selectivity was calculated on a molar carbon basis.

Supplementary Table 5 Computed adsorption energies (E_{ad}) of reaction intermediates

Catalyst	Adsorbate	Adsorption energy (eV)
CdS(100)	$\bullet CH_2OH$	-0.2
	$CH_3O\bullet$	-0.1
ZnS(100)	$\bullet CH_2OH$	-0.5
	$CH_3O\bullet$	-0.6
TiO_2(110)	$\bullet CH_2OH$	-1.3
	$CH_3O\bullet$	-1.1
CuS(001)	$\bullet CH_2OH$	-1.0
	$CH_3O\bullet$	-1.7

Supplementary Table 6 Control experiments with different reactants over the MoS_2-foam/CdS catalyst

Reactant*	Time (h)	Product amount† (mmol)								e^-/h^+	Selectivity‡ (%)
		EG	HCHO	HCOOH	GLD	OX	CO	CO_2	H_2		EG
CH_3OH	6	0.61	0.15	0	0	0	0	0	0.70	0.9	89
CH_3OH	12	1.3	0.30	0	0	0	0	0	1.4	0.92	90
CH_3OH	24	2.0	0.69	0.025	0.12	0.0055	0	0	2.8	0.94	80
CH_3OH	48	2.4	1.7	0.075	0.27	0.015	0	0	5.5	1.1	67
EG	12	-	0	0.046	0.28	0.12	0.049	0.076	1.5	1.3	-
EG	48	-	0.31	0.22	0.96	0.44	0.28	0.41	5.8	1.2	-

Reaction conditions: catalyst, 10 mg; solution, 76 wt% CH_3OH + 24 wt% H_2O or 1.0 M EG aqueous solution, 5.0 cm^3; atmosphere, N_2; light source, 300 W Xe lamp, visible light (λ = 420-780 nm). *Methanol: 76 wt% CH_3OH + 24 wt% H_2O; EG: 1.0 M EG. †GLD: glycoladehyde; OX: oxalic acid. ‡The selectivity was calculated on a molar carbon basis.

Supplementary Methods

Materials and catalysts

For the synthesis of CuS[1], 12 mg of L-cysteine was added into the 6 mL of water, and then 50 μL of 1 M $CuCl_2$ aqueous solution was added into the above L-cysteine solution. The resulting mixture was aged in an autoclave at 160 ºC for 12 h. The product was centrifuged and washed. For the synthesis of Bi_2S_3[2], 0.2 mmol $Bi(OAc)_3$, 1.5 mmol oleic acid and 5 ml octadecene were added in a flask and purged with Ar gas. Then, the temperature of reaction mixture was increased to 150 ºC and annealed for 30 min to dissolve $Bi(OAc)_3$. Subsequently, sulfur stock solution was injected to reaction mixture at 170 ºC and annealed for 30 min. The sample was collected and washed. For the synthesis of Cu_2O[3], CuCl was hydrolysed by adding an aqueous solution of Na_3PO_4 (1.0 M, 40 mL) into a NaCl aqueous solution (5.0 M, 400 mL) containing CuCl (0.04 mol) under vigorous stirring and an Ar flow. The obtained powder was washed with distilled water, followed by drying in vacuum. Cu_2O powder was obtained by heating at 400 °C for 24 h in vacuum. For the synthesis of ZnS, aqueous solutions of $ZnSO_4$ and Na_2S with concentrations of 0.1 M were mixed together, resulting precipitation. The precipitate was recovered by filtration, followed by washing with distilled water and drying at 60 ºC for 12 h. For the synthesis of g-C_3N_4, typically, 10 g of urea powder was put into an alumina crucible with a cover and then heated to 550 °C at a rate of 0.5 ºC /min in a muffle furnace and maintained at this temperature for 3 h. g-C_3N_4 was obtained after cooling down to room temperature. For the synthesis of ZnO[4], 0.5 M H_2O ethanol solution (150 mL) was added dropwise to a 0.2 M $Zn[OC(CH_3)_3]_2$ hexane solution (100 mL) under argon atmosphere at 25 ºC. ZnO powder was obtained by centrifugation and drying at 60 ºC for 4 h. TiO_2 (P25), which contained 20% rutile and 80% anatase, was purchased from Degussa.

For the synthesis of CdS nanoparticle[5], an aqueous solution of Na_2S was added slowly to $CdCl_2$ solution under stirring with the molar ratio of 1.2:1. The resulting slurry was filtered. The wet solid was suspended in deionized water (60 mL) and was transferred to a Teflon-lined autoclave (100 mL), followed by thermal treatment at 200 ºC for 24 h. After that, the yellow solid was filtered, washed with deionized water and ethanol, and then dried at 60 ºC. For the synthesis of nanosphere[6]: 1.6 mmol of $Cd(CH_3COO)_2 \cdot 2H_2O$ and 40 mmol of CH_4N_2S were dissolved in 40 mL of deionized water. Then, the solution was transferred into a Teflon-lined autoclave and was

subjected to hydrothermal treatment at 140 ºC for 5 h. The solid was obtained by centrifugation and washed with deionized water and ethanol, followed by drying at 60 ºC. For the synthesis of nanosheet[7], $CdCl_2 \cdot 2.5H_2O$ (0.0732g), S powder (0.064g) and diethylenetriamine (DETA, 12 mL) were added into a Teflon-lined autoclave, and was subjected to hydrothermal treatment at 80 ºC for 48 h. The solid was collected by centrifugation and washed with ethanol and distilled water, followed by drying at 40 ºC for 6 h to obtain a CdS-DETA. Then, the CdS-DETA (20 mg), L-cysteine (10 mg), DETA (0.1 mL) and H_2O (40 mL) were added into a beaker and were sonicated continuously for 2 h. The resultant solid were centrifuged and collected.

Preparation of Pt/CdS, Pd/CdS and NiO_x/CdS. Pt, Pd and NiO_x were loaded onto CdS nanorods by a photoreduction technique. In brief, Pt particles were deposited by photoreducing H_2PtCl_6 in an aqueous solution containing methanol as a sacrificial agent under irradiation with a 300 W Xe lamp for 1 h. $PdCl_2$ and $NiCl_3$ were used as the precursors instead of H_2PtCl_6 for the preparation of Pd/CdS and NiO_x/CdS via photodeposition of Pd and NiO_x, respectively.

Characterization

Scanning electron microscopy (SEM) measurements were carried out using a ZEISS SIGMA scanning electron microscope with 20 kV accelerating voltage. Transmission electron microscopy (TEM) measurements were performed on a Phillips Analytical FEI Tecnai 30 electron microscope operated at an acceleration voltage of 300 kV. The high-angle annular dark-field scanning transmission electron microscopy (HAADF-STEM), energy-dispersive X-ray spectroscopy (EDX) mapping and 3D tomography were carried out on the FEI Talos F200X microscope operated at 200 kV. N_2 physisorption was carried out with a Micromeritics Tristar 3020 surface area and porosimetry analyzer. Diffuse reflectance Ultraviolet-visible (UV-vis) spectroscopic measurement was performed on Varian-Cary 5000 spectrophotometer equipped with a diffuse reflectance accessory. The spectra was collected with $BaSO_4$ as a reference.

The steady-state photoluminescence (PL) spectroscopic measurements were performed with Hitachi F-7000 fluorescence spectrophotometer at an excitation wavelength of 405 nm. The time-resolved photoluminescence (TRPL) spectra were recorded on a Horiba Jobin Yvon FluoroMax-4 spectrofluorometer at an excitation wavelength of 405 nm. The extended X-ray absorption fine structure (EXAFS)

spectroscopic measurements were carried out at the BL14W1 beamline of the Shanghai Synchrotron Radiation Facility (SSRF).

Photoelectrochemical measurements were carried out with CHI 760E using a standard three-electrode cell with a working electrode, a Pt wire as the counter electrode, and an SCE electrode as the reference electrode. A 0.5 M solution of Na_2SO_4 was used as the electrolyte. The working electrode was prepared by cleaning an F-doped SnO_2-coated glass (FTO glass, 1 cm × 2 cm). The photocatalyst was dispersed in ethanol, and the suspension was added dropwise directly onto the FTO by microsyringe with a gentle stream of air to speed drying. The film was dried at 80 °C for 1 h, and the typical surface density of the photocatalyst was 1 mg cm^{-2}.

The linear sweep voltammetry (LSV) curves measurements were carried out with CHI 760E. The electrode preparation process was as following: 4 mg catalyst and 4 mg carbon black were suspended in 1 ml ethanol with 20 μL Nafion solution (5 wt.%, Du Pont) to form a homogeneous ink assisted by ultrasound. Then 25 μL of the ink was spread onto the surface of glassy carbon. LSV tests were conducted in an Ar-saturated 0.5 M H_2SO_4 electrolyte.

Electron spin resonance (ESR) spectroscopic measurements were performed at room temperature using a Bruker EMX-10/12 ESR spectrometer operated at X-band frequency. The parameters for ESR measurements were as follows: microwave frequency 9.8 GZ, microwave power 20 mW, modulation frequency 100 kHz, attenuator 10 dB. For in situ ESR measurements, CdS or MoS_2-foam/CdS powders were dispersed in a mixed solution of 76 wt% CH_3OH and 24 wt% H_2O containing DMPO (0.080 M), which was used as a spin-trapping agent, by ultrasonic treatment. Then, the suspension was injected into a glass capillary and the glass capillary was placed in a sealed glass tube under N_2 atmosphere. The sealed glass tube was placed in the microwave cavity of ESR spectrometer and was irradiated with Xe lamp (λ = 420-780 nm) during ESR measurements at room temperature.

Computational method

Computational setup. The rutile TiO_2(110) surface was modeled by a five O-Ti-O trilayer slab with the xy dimensions of 4×2 supercell, as shown in Supplementary Fig. 17a. For hexagonal wurtzite CdS(100), a 3×2 unit cell was used with a slab thickness of four bi-layers, as depicted in Supplementary Fig. 17b. Lattice constants of

a = b = 4.669 Å and c = 2.97 Å for TiO_2 and a = b = 4.15 Å and c = 6.737 Å for CdS were used. For sphalerite ZnS(100), a 3×2 unit cell was used with a slab thickness of 4 bi-layers, and the lattice constants of a = b = 3.851 Å and c = 6.312 Å were used. For hexagonal CuS(001), a 4×3 unit cell was used with a slab thickness of 11 layers, and the lattice constants of a = b = 3.797 Å and c = 16.44 Å were used. The slabs were separated by a vacuum space of 20 Å, and full 3D periodic boundary conditions were applied. The thickness of slabs was investigated to ensure convergence on band positions and water adsorption energies. The slabs were covered by one monolayer of water molecules on both sides of the slabs. In calculating the dehydrogenation energies of CH_3OH, one adsorbate molecule was placed on the TiO_2 and CdS surface, equivalent of a coverage of 1/8 ML on TiO_2 and 1/6 ML on CdS. The bottom two layers were fixed during geometry optimization.

Density functional theory (DFT) calculations were carried out using the freely available program package CP2K/Quickstep[8]. The 2s, 2p electrons of the O, C and S atoms and the 3s, 3p, 4s, 3d electrons of the Ti and Cd atoms were treated as valence electrons. The basis sets were short-ranged (less diffuse) double-ζ basis functions with one set of polarization functions (DZVP)[9]. We also tested the adsorbed molecules in gas phase using larger basis sets (TZV2P) and found a negligible difference of 0.01 eV in the adsorption energies. The plane wave cutoff for the electron density is 400 Ry. The core electrons were represented by analytic Goedecker-Teter-Hutter (GTH) pseudopotentials[10]. We used the GGA-PBE functional, and also the hybrid HSE06 for accurate description of the hole states[11, 12]. The Grimme's dispersion correction was employed[13]. For hybrid functional calculations, the auxiliary density matrix method (ADMM) recently developed and implemented in CP2K[14, 15], was used to re-expand the density matrix with small auxiliary basis sets, leading to great speed-up of the calculation of Hartree-Fock exchange (HFX). The transition states for the concerted proton electron transfer reactions of the C-H/O-H breaking in CH_3OH on CdS were searched on ground state potential energy surfaces using the climbing-image nudged elastic band (CI-NEB) method. Detailed analyses of the transition states will be reported elsewhere.

Computation of concerted proton-electron transfer energies. A method for computation of free energies of concerted proton-electron transfer (CPET) reactions developed by Cheng, Sprik and co-workers, combines density functional theory based

molecular dynamics (DFTMD) and free energy perturbation theory to comprehensively account for electronic structures of solutes/surfaces and solvents and all entropic contributions to free energies[16]. It is however rather expensive, and thus a simplified scheme using total energies of optimized structures has been developed and validated on TiO_2, showing a reasonable accuracy[17]. We summarize a few key points in the following, and refer to the previous publications for detail.

The PT-ET and CPET reactions are shown in a triangular form in Supplementary Fig. 17c. The dehydrogenation energy $\Delta_{dh}G^o$ (diagonal) must be equal to the sum of the deprotonation energy $\Delta_{dp}G^o = 2.3\mathrm{k_B}T\mathrm{p}K_\mathrm{a}$ (horizontal) and oxidation energy $\mathrm{e}U^o$ (vertical), as required by the Hess's Law. The oxidation energy and the dehydrogenation energy are referenced to the standard hydrogen electrode (SHE). The dehydrogenation energy can be approximately expressed as follows,

$$\Delta_{dh}G^o_{XH} = \Delta_{dh}E_{XH} - \mu^{g,o}_{H^+} - \Delta_{zp}E_{H(X)} + \mathrm{k_B}T\ln\left[\mathrm{c^o}\,\Lambda^3_{H^+}\right] \quad (5)$$

in which $\Delta_{dh}E_{XH}$ is the dehydrogenation total energy of XH obtained from static DFT calculations. $\mu^{g,o}_{H^+}$ is the standard chemical potential of a gas phase proton, and we use the experimental value of 15.81 eV. In addition, two correction terms, $\Delta_{zp}E_{H(X)}$ and $\mathrm{k_B}T\ln\left[\mathrm{c^o}\,\Lambda^3_{H^+}\right]$ ($\mathrm{c^o}$ is the standard concentration 1 mol L^{-1}, and Λ_{H^+} is the thermal wavelength of the proton), are included for correction of the zero point energy of X-H and translational entropy of a proton at the standard concentration, respectively. Their values are 0.35 eV and -0.19 eV, respectively. The derivation of Eq. 5 and the physical meanings of the terms have been explained in more detail in our previous publications[17, 18]. The deprotonation energy can be also calculated using the simplified scheme in which the pK_a difference between surface species and a reference surface site is estimated from the total energy difference of the acid-base reaction on the surface with the adsorption of one ML water. Applying the Hess's Law, the oxidation energy can thus be obtained by subtracting the deprotonation energy from the dehydrogenation energy.

To obtain accurate energies in Supplementary Fig. 17c, it is important to ensure the calculated intermediates have the correct (localized) spin states. The PBE functional tends to give delocalized states due to the well-known delocalization error at the GGA level. The hybrid functional therefore is critical to correctly describe the electronic states of radical intermediates. We were aware of this issue and the reported results in this work were obtained with screened hybrid HSE06.

Details of DFT calculations. The adsorption structures of methanol on TiO_2 and CdS are shown in Supplementary Fig. 17a to 17b. Adsorbates are placed at both sides of the slabs to minimize the internal dipoles within the slabs. Adsorbed methanol binds with its O atom on the Ti site on TiO_2 and on the Cd site on CdS. The optimized structures of the most stable configurations of the radical intermediate states are shown in Supplementary Fig. 17d to 17g, and the visualization of spin densities confirms that the spin states have been correctly localized on the radicals CH_2OH• and CH_3O•. The computed adsorption energies of the reaction intermediates, using the hybrid HSE06 functional are listed in Supplementary Table 5.

The computed energies of the proton and electron transfer steps in methanol oxidation on rutile TiO_2 (110) and CdS(100) are shown in the Supplementary Fig. 17h. The dehydrogenation energies were calculated from total energy differences using Eq. (5). The deprotonation energies on TiO_2 were obtained using the simplified scheme described above. Since the adsorption on CdS is very weak, it is expected that the deprotonation energies of methanol would be close to those in aqueous solution. Thus, the experimental p*K*a's in solution are used to estimate the deprotonation energies on CdS. Note that they should set the upper bounds of the estimates considering the adsorption may weaken C-H/O-H bonds, leading to lower p*K*a's. The oxidation energies are calculated by taking the differences between the above calculated dehydrogenation and deprotonation energies. The energetics of the proton and electron transfer steps in Supplementary Fig. 17h are used to calculate the energies in the reaction profile of Fig. 2c in the main text. Note that the energies shown in Fig. 2c in the main text also accounts for the contributions from the photo-excited holes.

Supplementary References

1. Zhang, Y. *et al*. Biomolecule-assisted, environmentally friendly, one-pot synthesis of CuS/reduced graphene oxide nanocomposites with enhanced photocatalytic performance. *Langmuir* **28**, 12893-12900 (2012).
2. Manna, G., Bose, R. & Pradhan, N. Photocatalytic Au-Bi_2S_3 heteronanostructures. *Angew. Chem. Int. Ed.* **53**, 6743-6746 (2014).
3. Hara M. *et al*. Cu_2O as a photocatalyst for overall water splitting under visible light irradiation. *Chem. Commun.* 357-358 (1998).
4. Li, D. & Haneda, H. Morphologies of zinc oxide particles and their effects on photocatalysis. *Chemosphere* **51**, 129-137 (2003).
5. Gao, W., Xu, Y., Chen, Y. & Fu, W. Highly efficient and selective photocatalytic reduction of nitroarenes using the Ni_2P/CdS catalyst under visible-light irradiation. *Chem. Commun.* **51**, 13217-13220 (2015).
6. Lin, G., Zheng, J. & Xu, R. Template-free synthesis of uniform CdS hollow nanospheres and their photocatalytic activities. *J. Phys. Chem. C* **112**, 7363-7370 (2008).
7. Xu, Y., Zhao, W., Xu, R., Shi, Y. & Zhang, B. Synthesis of ultrathin CdS nanosheets as efficient visible-light-driven water splitting photocatalysts for hydrogen evolution. *Chem. Commun.* **49**, 9803-9805 (2013).
8. VandeVondele, J. *et al*. Quickstep: Fast and accurate density functional calculations using a mixed Gaussian and plane waves approach. *Comput. Phys. Commun.* **167**, 103-128 (2005).
9. VandeVondele, J. & Hutter, J. Gaussian basis sets for accurate calculations on molecular systems in gas and condensed phases. *J. Chem. Phys.* **127**, 114105 (2007).
10. Hartwigsen, C., Gœdecker, S. & Hutter, J. Relativistic separable dual-space Gaussian pseudopotentials from H to Rn. *Phys. Rev. B* **58**, 3641-3662 (1998).
11. Heyd, J., Scuseria, G. E. & Ernzerhof, M. Hybrid functionals based on a screened Coulomb potential. *J. Chem. Phys.* **118**, 8207-8215 (2003).
12. Krukau, A. V., Vydrov, O. A., Izmaylov, A. F. & Scuseria, G. E. Influence of the exchange screening parameter on the performance of screened hybrid functionals. *J. Chem. Phys.* **125**, 224106 (2006).
13. Kresse, G. & Furthmüller, J. Efficiency of ab-initio total energy calculations for metals and semiconductors using a plane-wave basis set. *Comp. Mat. Sci.* **6**, 15-50 (1996).
14. Guidon, M., Schiffmann, F., Hutter, J. & VandeVondele, J. Ab initio molecular dynamics using hybrid density functionals. *J. Chem. Phys.* **128**, 214104 (2008).
15. Guidon, M., Hutter, J. & VandeVondele, J. Auxiliary density matrix methods for Hartree-Fock exchange calculations. *J. Chem. Theory Comput.* **6**, 2348-2364 (2010).
16. Cheng, J., Liu, X., VandeVondele, J., Sulpizi, M. & Sprik, M. Redox potentials and acidity constants from density functional theory based molecular dynamics. *Acc. Chem. Res.* **47**, 3522-3529 (2014).

17. Cheng, J., Liu, X., VandeVondele, J. & Sprik, M. Reductive hydrogenation of the aqueous rutile TiO_2(110) surface. *Electrochim. Acta* **179**, 658-667 (2015).
18. Cheng, J., Liu, X., Kattirtzi, J. A., VandeVondele, J. & Sprik, M. Aligning electronic and protonic energy levels of proton-coupled electron transfer in water oxidation on aqueous TiO_2. *Angew. Chem. Int. Ed.* **53**, 12046-12050 (2014).
